# Work schedule and seasonal influences on sleep and fatigue in helicopter and fixed-wing aircraft operations in extreme environments

**DOI:** 10.1038/s41598-022-08996-2

**Published:** 2022-05-18

**Authors:** Adam Fletcher, Simon Stewart, Karen Heathcote, Peter Page, Jillian Dorrian

**Affiliations:** 1 University of South Australia, UniSA Justice and Society, Adelaide, 5001 Australia; 2 Occupational Alertness and Fatigue Management, Integrated Safety Support, Melbourne, 3000 Australia; 3 Babcock International Group, Aviation, 33 Wigmore, London, W1U 1QX UK; 4 Sirius Aviation Limited, The Saint Botolph Building, 138 Houndsditch, London, EC3A 7AR UK; 5 Fatigue Risk Management Software and Decision Support, InterDynamics, Brisbane, 4000 Australia

**Keywords:** Neuroscience, Physiology, Psychology, Health occupations, Risk factors

## Abstract

Sleep and fatigue were investigated in aviation search and rescue, firefighting, emergency medical services and offshore transfer operations in 210 participants, for 21 days each, across 17 datasets in seven countries. Sleep data were collected using wrist-worn actigraphs and sleep diaries. Sustained attention was assessed using a 5-min Psychomotor Vigilance Task (PVT). Duty information was provided from corporate IT systems. Despite the number of 24 h operations, most work occurred during daytime hours, and most sleep occurred at night. There were seasonal changes in work and sleep patterns, with naps used to augment total sleep time. The proportion of sleep occurring during duty varied from zero to 30%. Differences in PVT response times were trivial to small. Legislation that defines flight, duty time and minimum rest limits assume that sleep is not obtained during duty periods, apart from some napping under Fatigue Risk Management Systems (FRMS). However, especially in cases where the aviation service requires waiting for tasks (e.g. search and rescue, emergency medical response), this assumption may not always hold. FRMS should accommodate different modes of working that safely facilitate sleep during duty time where appropriate.

## Introduction

Scientific investigation of work hours, fatigue, and performance has been occurring since the early 1890s. Initial studies focused on physical aspects of performance and productivity in industrial settings such as factories, mines, and workshops^1,2^. After World War 1 began in 1914 there was a reactive wave of focus on industrial health and fatigue, particularly documented in Britain and parts of Europe^3^. At that time, soldiers, medical staff, transport workers, and other groups were working long and varied hours often in extreme environments. However, most available studies were conducted in more controlled work settings such as ammunition factories where measurable health, productivity and safety issues were being recorded in detail^2,4^. Around the same time, military aircraft were being used by both sides in the war, for offensive, defensive, and reconnaissance missions. Again, operational hours could be long and varied, and conditions often extreme. Research into sleep loss and fatigue in these environments, however, did not start appearing in written form until the late 1930s^5^. After World War 2 the published scientific research on fatigue began to focus on commercial aviation through the 1950s and 1960s, including investigation of the effects of time zone changes on daily (i.e. circadian) time clocks^6–8^.

Sleep as a factor in commercial aviation safety research expanded through the 1970s, 1980s, and beyond. Aspects investigated include East-West time zone travel^9–12^, bio-chemical markers and impacts^13,14^, differences between shorter- and longer-haul flying operations^15–19^, and personal management approaches^20^. Sleepiness and fatigue are overlapping constructs, with definitions that have received attention in the scientific literature, and have been at the centre of much debate^21,22^. From an industry perspective, the word ’fatigue’ is more frequently used, being defined in aviation environments as, “*a physiological state of reduced mental or physical performance capability resulting from sleep loss or extended wakefulness, circadian phase, or workload (mental and/or physical activity) that can impair...alertness and ability to safely operate an aircraft or perform safety-related duties*”^23^ (page xii). We follow this conceptualisation as our working definition of fatigue used in this paper, in contrast to sleepiness, which we are using to refer to the drive for sleep^21^. Collectively, there are hundreds of published research articles spanning work hours, sleep, alertness, fatigue, and performance issues in military and commercial aviation settings. In contrast, there are safety-critical sectors within the aviation industry that have received less systematic attention.

More specifically, there are both rotary-wing (i.e. helicopter) and fixed-wing (i.e. aeroplane/airplane) sectors that have unique characteristics that may result in vulnerability to fatigue, despite the fact that the majority of operations are during daylight hours, and that there may be no requirement for timezone crossing^24^—two focus sources of fatigue from the broad commercial and military aviation literature. Indeed, operations such as emergency medical services, firefighting, search and rescue, and transport of people and equipment to and from offshore facilities often require significant use of stand-by since a key feature of these services is being ready to fly for low-probability events. These services also tend to have very intense and very quiet seasons^25^—the extreme example is firefighting, which is busy in summer and may not be required at all during other parts of the year. These features of the work, alongside the 24 h operations where workers may sleep on base^25^, result in a situation whereby workers have opportunities to sleep during and between duty periods, in various sleeping accommodations.

Together, key sources of fatigue for these unique environments include multiple flights in a duty period, extended duration flights, and many consecutive duty periods. On-call operations (or stand-by) may be unpredictably punctuated by high tempo, safety-critical activity. There may also be taxing environmental conditions (e.g. high vibration levels, low or high temperatures), exposure to distressing events, and seasonal peaks and troughs, including duty lengths that may depend on sunlight hours^24–28^. Work environments vary substantially between countries and operators^25^. These key sources of fatigue are often poorly reflected in flight and duty regulations for pilots. Other safety-critical roles including engineers, medical staff, rescue swimmers and other mission crew are more rarely considered in aviation safety legislation compared with pilots. Feasible options for managing sleep- and fatigue-related risks may be very different to those applicable in scheduled commercial aviation including passenger airline operations^25^.

The primary aim of this research study, which was performed in a real-world (i.e. naturalistic) context was to measure key sleep, work, alertness, mental performance and other fatigue-related data within several aviation emergency medical, firefighting, search and rescue, offshore transport, and other mission-critical contexts. Our meaning of “extreme environments” is that these services often work with demanding geographies (e.g. mountains, open ocean), weather, and tasks (e.g. rescues, vehicle crashes). In this investigation, we describe and examine the patterns of work and sleep for personnel during duty days and non-duty days, examining differences in work and sleep during peak (e.g. summer) and off-peak (e.g. winter) seasons. We also assess differences in sleep and performance as a function of work factors (time of day, number of consecutive days of duty, role type, location and mission type.

## Methods

### Participants, studies, and datasets

Participants (n = 210) were employed by a company providing rotary and fixed wing emergency medical services (EMS), search and rescue (SAR), and oil and gas services across >90 operating bases (94% male, 25–30 y = 7%, 30–40 y = 28%, 40–50 y = 43%, 50–60 y = 21%, 60 + y = 2%). The sample included pilots (77%, rotary wing = 64%, fixed wing = 13%), other crew (9%) and technicians (14%), across seven countries (France = 11%, Italy = 41%, Spain, Portugal = 28%, Sweden, Finland = 9%, UK = 11%), in emergency medical (70%), search and rescue (9%), firefighting (14%), and air transfers (7%).

Data were collected between November 2014 and December 2018, over 21-days for each study, during which, participants continued usual duties, except to complete study questionnaires and performance tasks. In this manuscript, consistent with the language used in these operational environments, ‘duty periods’ will be used to refer to work shifts, and ‘duty days’ and non-duty days’ will be used to refer to work days and days off, respectively. Experimenters visited each operating company (opco) to provide equipment and training. The individual worksites were visited by experimenters or trained local project managers to provide instructions, and to distribute research materials and consent forms. Local project managers supported data collection, while experimenters remained contactable. The UniSA Human Research Ethics Committee exempt this project under clauses 5.1.22 and 5.1.23 of the National Statement^29^, participants gave documented informed consent for the data to be published, and all methods were performed in accordance with the relevant guidelines and regulations. Studies yielded a total of 17 datasets (Table 1). Participants were on duty at base, working on-call (response) or continuous (scheduled tasks) modes. Emergency medical and search and rescue operations were run around-the-clock, whereas firefighting and transfers were daytime operations. For the first four studies in Italy, pilots and technicians participated in the study twice, during off-peak (November, January) and peak (June, July) periods.

**Table 1:** Summary information for each of the datasets, presented in chronological order.

#	Location	Month (start)	Year	Daylight (hh:mm)	Type	Wings	Roles	Mode	24 h	Consecutive days median, max	PVT	Sleep quality
1	Italy (off-peak)	Nov	2014	10:13	EMS	Rotary	Pilots	On-Call	Y	4, 12	Y	
2	Italy (peak)	Jun	2015	15:06	EMS	Rotary	Pilots	On-Call	Y	4, 8	Y	
3	Italy (peak)	Jul	2015	15:05	EMS	Rotary	Tech	Continuous	Y	2, 8	Y	
4	Italy (off-peak)	Jan	2016	09:15	EMS	Rotary	Tech	Continuous	Y	5, 15	Y	
5	Sweden, Finland	Apr	2016	14:00	EMS	Rotary	Pilots	On-Call	Y	2, 8	Y	
6	Sweden, Finland	Apr	2016	14:00	EMS	Fixed	Pilots	On-Call	Y	2, 5	Y	
7	Italy	Aug	2016	14:10	Fire	Fixed	Pilots	Continuous	N	3, 6	Y	
8	UK	Jan	2017	08:00	EMS	Rotary	Pilots	On-Call	Y	2, 4	Y	
9	Spain	Mar	2017	11:30	EMS	Fixed	Pilots	On-Call	Y	4, 11	Y	
10	Spain	Mar	2017	11:30	EMS	Rotary	Pilots	On-Call	Y	1, 5	Y	
11	Portugal	May	2017	14:00	EMS	Rotary	Pilots	On-Call	Y	1, 3	Y	
12	UK	Feb	2018	09:20	Transfers	Rotary	Pilots	Continuous	N	3, 8		Y
13	France	May	2018	14:50	EMS	Rotary	Pilots	On-Call	Y	4, 12		Y
14	Spain	Aug	2018	14:10	Fire	Rotary	Pilots	Continuous	N	9, 24		Y
15	Spain	Aug	2018	14:10	Fire	Rotary	Crew	Continuous	N	8.5, 23		Y
16	Spain	Sep	2018	12:55	SAR	Fixed	Pilots	On-Call	Y	1.5, 5		Y
17	Spain	Sep	2018	12:55	SAR	Fixed	Crew	On-Call	Y	4, 11		Y

Datasets are shown by geographic location (Location), start month (Month) and year (Year) of data collection, daylight hours at that location at that time of year (Daylight), Type of Operation (Type), whether the operation involved fixed or rotary (helicopter) wing aircraft (Wings), participant job roles (Roles), whether the operation involved continuous or “on-call” work (Mode), whether the operation ran across the full 24 h (24 h), the median and maximum number of consecutive days of duty in each dataset (Consecutive Days), whether PVT was collected (PVT), and whether Sleep Quality ratings were collected (Sleep Quality).

EMS, emergency medical service; Fire, firefighting; Transfers, Air Taxi; SAR, search and rescue; Tech, technicians; Y, yes; N, no.

### Measures

Participants wore activity monitors (Philips Respironics Actiwatch 2, Oregon, USA) and completed either electronic tablet-based work and sleep diaries (datasets 1–11) or paper-based sleep diaries, with duty and flight information provided directly from opco’s electronic system (datasets 12–17). Also delivered on the tablets (datasets 1–11) was a version of the Psychomotor Vigilance Task, which participants were instructed to complete during breaks in duty periods and on days off.

#### Sleep, wake, and work hours

For each sleep period, including naps, participants provided sleep/wake times and dates, and for datasets 12–17, participants completed sleep quality ratings (1 = Very Good, 2 = Good, 3 = Average, 4 = Poor, 5 = Very Poor). Activity monitors (with Actiware-sleep software, Cambridge Neurotechnology Ltd.) are wristwatch-like devices containing a piezo-electric accelerometer that measures movement $>\,0.1$ g, sampling every 125 ms and storing data in 1-min intervals. Movement data are combined with diary data (using diary-recorded time-in-bed data to set sleep intervals for analysis^30^) and a standard algorithm, validated against polysomnographic sleep measures^31–34^, used to estimate sleep time. Daily duty records (tablet-based diaries, Air Maestro Flight and Duty App, Avinet, Australia, or opco electronic system records), included start and end dates and times and breaks. Duty records were compared to sleep diaries. When two consecutive duty records abutted, or were within 45 min of each other, they were merged into a single period. Active duty data (flight or other activity) was examined relative to sleep period data. Since sleep could occur during duty hours, but active duty could not occur during sleep periods, where an overlap existed, duty time around the sleep time was truncated to create corrected on-duty times. Variables extracted included total 24 h sleep time (from midday-to-midday, since most sleep periods were across the nighttime hours), total sleep time in the 24 h prior to duty (anchored to duty start time), total sleep time in the 48 h prior to duty, and total wake time (number of consecutive hours awake) at the end of duty.

#### Psychomotor vigilance task (PVT)

The PVT is a widely used response-time task requiring sustained attention^35^, with validated versions differing in delivery mechanism and duration^36–38^. Participants watched a screen for a 5-min, during which, a stimulus (a millisecond counter) appeared periodically with an inter-stimulus interval varying randomly from 1 to 5 s. The participant pressed a button with the thumb of their dominant hand as quickly as possible in response to each stimulus, which stopped the counter, displaying their response time (RT) in milliseconds. Average RT across the stimuli in ms is reported for this study. As this was a naturalistic study, PVT trial locations may have included distractions such as in shared kitchens, crew rooms or hangars. Therefore, mean response times can be expected to be longer than in highly controlled laboratory environments.

### Data processing and analysis

**Figure 1:**
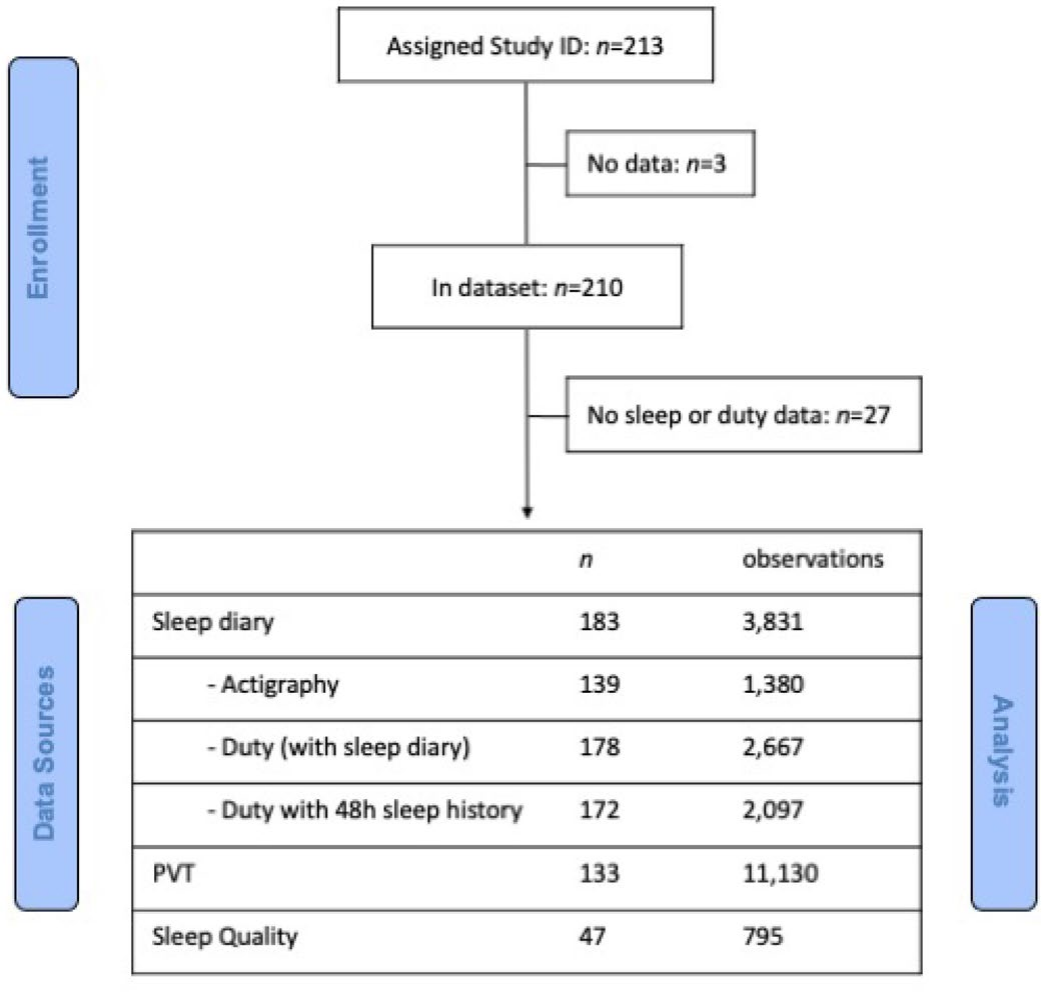
Consort diagram. Data flow from participant enrolment, through to number of observations for analysis for each variable. *Note:* Observations for Actigraphy, Duty (with sleep diary), and Duty (with 48 h sleep history) are nested within the Sleep diary sample. Sample sizes provided for PVT and Sleep Quality are independent. PVT=Psychomotor Vigilance Task.

The final dataset included 210 participants, with data collected over 3204 discrete days, yielding 3090 sleep periods, 2707 duty periods, and 11,130 PVT trials (Fig. 1). Where there were no data for a particular participant (*n* = 3) or there were either no sleep or duty data (*n* = 27) then that individual was excluded from analyses.

#### Sleep diaries and actigraphy

**Figure 2:**
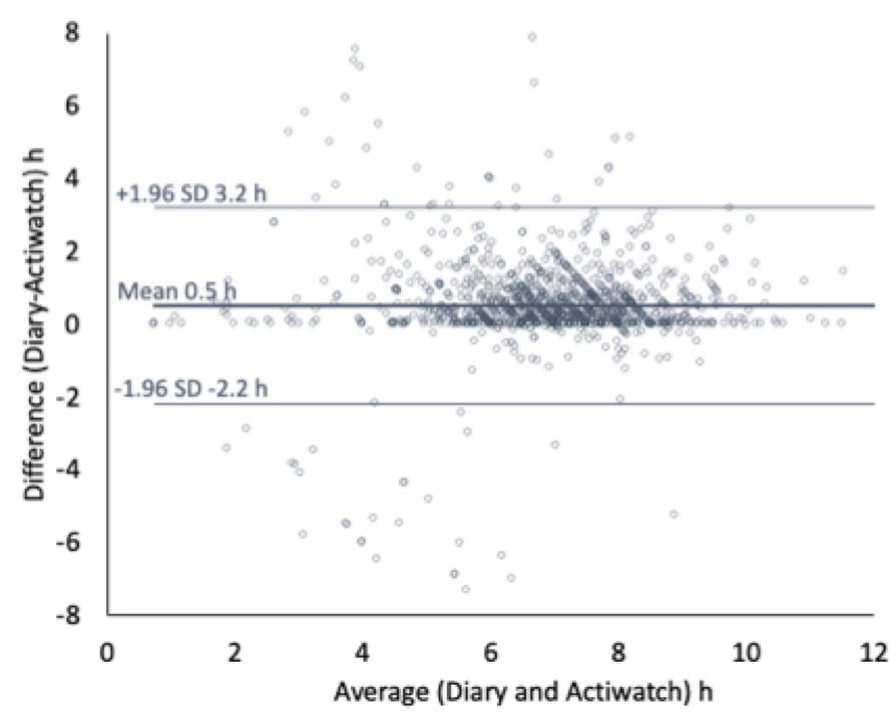
Bland–Altman Plot. Illustrates the relationship between Diary and Actigraphy estimates of sleep in the 24 h prior to starting duty. The mean difference (diff) and 95% Confidence Intervals are indicated by the horizontal lines. The self-reported (i.e., diary) sleep duration was on average, 30 mins longer than more objective (i.e., actigraphy) estimates.

Bland–Altman Analysis^39^ (using the blandr package in r^40^) revealed that on average, diary estimates of sleep in the 24 h prior to starting duty were 30 min longer than actigraphy estimates (Bias = 0.50 h, 95%CI: Lower = 0.42 h, Upper = 0.58 h) and that the differences were relatively consistent across magnitudes of sleep (Fig. 2). Technical difficulties with actigraphs led to data loss (Fig. 1). Research highlights the strength of the combined use of sleep diaries and activity monitors in the actigraphic estimation of sleep relative to the ’gold standard’ sleep measurement using electroencephalography^30,31,41^. Our data are consistent with previous findings highlighting the limitations of actigraphy due to data loss^41^, and that polysomnographic measures, actigraphs, and diaries provide total sleep time estimates that are close, on average, but with wide inter-participant variability^42^. In this study, we are focused more on patterns rather than absolute amounts of sleep. Nevertheless, since our actigraphy sleep estimates (the combination of diary and actigraph measures) were 30 mins lower than diary estimates, to be conservative, discrete diary sleep estimates were reduced by 30-min to bring them in line with the more objective, continuous, actigraphy-derived values. This conservative approach should be noted when considering the absolute values of sleep provided in the results section.

#### Patterns of work and sleep for participants during duty days and non-duty days

Data for each participant was expressed in 15-min intervals from midday-to-midday (since most sleep occurred during nighttime hours), indicating whether duty or sleep occurred in each interval. This was then expressed as a percentage of the total number of duty or sleep periods in each interval, and the 24 h distributions were smoothed ($\pm \,1$, 15-min interval) and double-plotted. In order to test differences in sleep on duty days compared to non-duty days linear mixed effects analysis of variance (ANOVA) specified total 24 h sleep time as a dependent variable with fixed effect of day (duty/non-duty) and a random effect of subjectID on the intercept.

#### Sub-group analysis: sleep quality differences in home and work environments

Sleep quality ratings (studies 12–17, Table 1) were analysed, with each sleep period identified as occurring at home, on base, or in a hotel/other work accommodation. Given the large variability in how respondents completed sleep quality scales, analyses were only conducted comparing environments that included repeated measurements for the same people on repeated occasions. This resulted in analyses for: (a) UK transfers (#12) comparing home (n = 152) and hotel (n = 20) sleep periods in 9 participants; (b) France emergency medical services (#13) comparing home (n = 195) and base (n = 143) sleep periods in 14 participants; and (c) Spain search and rescue (#16, #17) comparing home (n = 114) and base (n = 45) sleep periods in 8 participants, and home (n = 82) and hotel (n = 44) sleep periods in 6 participants. Linear mixed effects ANOVA specified sleep quality rating (1–5) as a dependent variable with a fixed effect of sleep environment (home/base, or home/hotel) and a random effect of subjectID on the intercept.

#### Sub-group analysis: peak versus off-peak

Double-plotted 24 h distributions of duty and sleep were created, as described above, for Italy Pilots (#1 = Off-Peak, #2 = Peak) and technicians (#3 = Peak, #4 = Off-Peak). For pilots and technicians separately, linear mixed effects ANOVA specified sleep in the 24 h prior to duty as a dependent variable with a fixed effect of season (Peak/Off-Peak) and a random effect of subjectID on the intercept. The total area under the curve for the sleep distribution figures (described above) was correlated (Pearson *r*) with total hours of sunlight per day (harvested from online records of sunlight hours by location).

#### Differences in sleep and performance as a function of work factors

**Figure 3:**
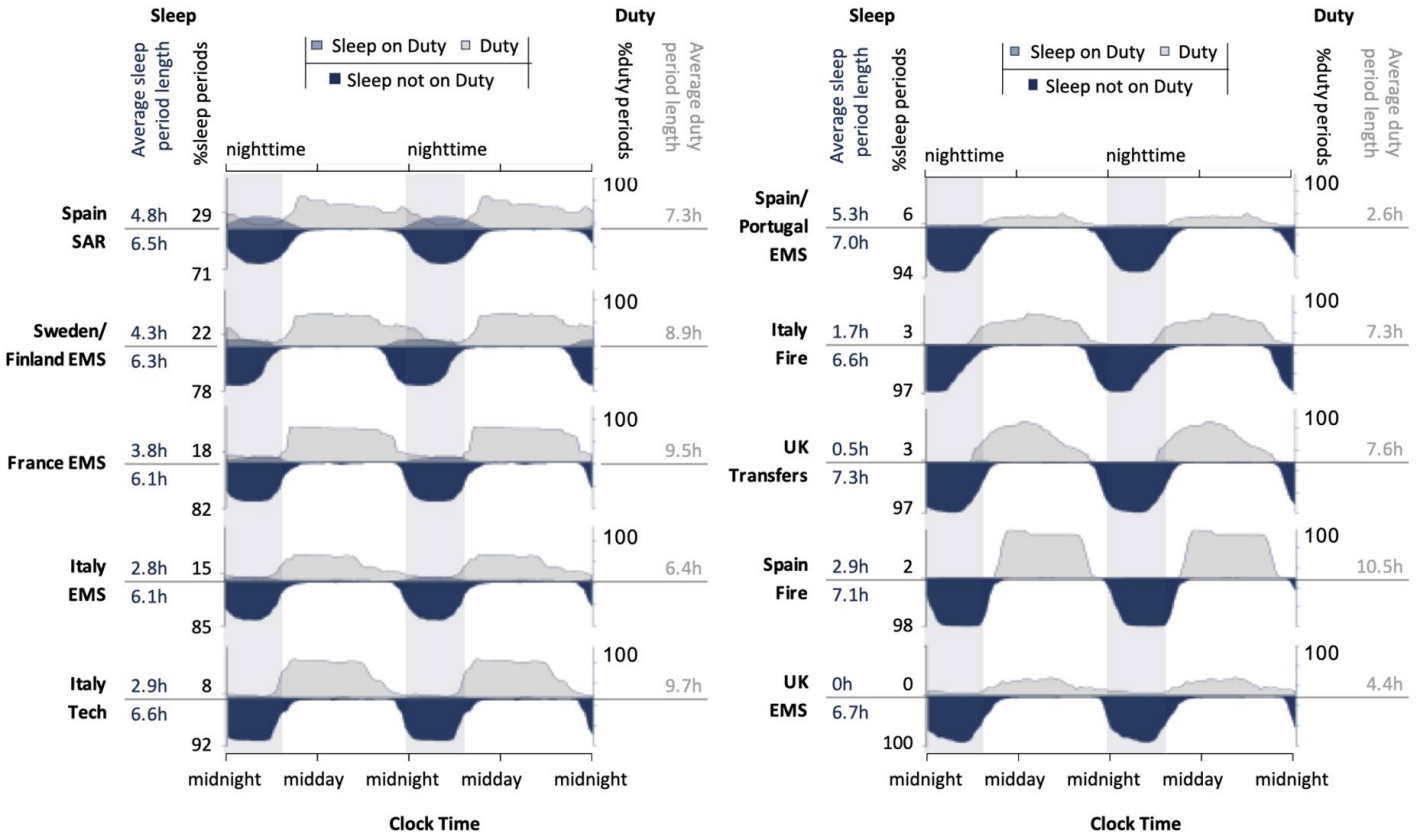
Work and sleep distributions vary as a function of location and type of operation. To characterise patterns of work and sleep, the 24 h distributions were double plotted. duty and sleep periods were expressed as a percentage of the total number of periods at that time of day (in 15-min bins). Sleep periods that were associated with duty were plotted, superimposed over duty, with sleep periods outside of duty also represented. *Note:* EMS = Emergency Medical Service, Fire = Firefighting, Transfers = Air Taxi, SAR = Search and Rescue, Tech = Technicians.

Since the majority of the work occurred during the day, to categorise the start times into cells of sufficient size for analysis, categories were: 0600–0759 h (27%), 0800–0959 h (36%), 1000–1659 h (18%), and 1700–0559 h (19%). Given the low number of data points and their spread across the nighttime hours, these four categories yielded the most even distribution of data points in each cell. This categorisation, while statistically motivated, is not optimal from a biological perspective, since there are important changes that occur across the night and into the circadian nadir^9^. The thin spread of nighttime observations (as can be seen in Fig. 3), did not allow for sub-categorisation. To compensate for this, we chose 0600–0759 h as our reference category in analyses. This limitation should be noted while considering findings presented in relation to start time. The number of consecutive duty days varied across studies (Table 1) and participants could begin data collection at any point in their work cycle. Therefore, we did not have the information to decide whether their first day of recorded data in the study was preceded by a day off, or by one or more duty days. We were only able to accurately classify consecutive days of duty in our dataset following a recorded day off. This led to the exclusion of the first several days of recorded data for this measure. To allow cells of sufficient size for analysis, categories were: first day after day off (22%), and subsequent shifts (78%). Other work factors included location, operation, and role (Table 1). Linear mixed effects models specified dependent variables of sleep in 24 h and 48 h prior to duty start, and total wake time at duty end, and Mean PVT RT. Fixed effects included location (France, Italy, Spain/Portugal, Sweden/Finland, UK, noting that in the PVT RT model, there were no data from France, and a reduced Spain and UK dataset, Table 1), operation (EMS/SAR, Fire/Transfers), duty start hour (0600–0759 h, 0800–0959 h, 1000–1659 h, 1700–0559 h), role (RW Pilot, FW Pilot, Other Crew, Technician), day (first duty day, subsequent duty day), and a role*day interaction term. All models included a random effect of subjectID.

#### Examining lowest sleep and highest wake quintiles

Distributions of sleep in the prior 24 h and 48 h and the distribution of prior wake (consecutive hours awake) at the end of duty across all participants and all duty periods were divided into quintiles. Membership of the lowest quintile of sleep in the prior 24 h and 48 h, and the highest quintile of prior wake at the end of duty (yes/no) were identified and the overlapping incidence of low sleep and high wake (i.e., a count of the low/high sleep/wake quintiles for each duty period, 0–3) was calculated. Generalised Estimating Equations for counts (negative binomial model) specified a dependent variable of sleep or wake quintile count (0–3) with predictors of location, operation, duty start hour, role, and day, with a panel variable of subject ID.

## Results

### Patterns of work and sleep for participants during duty and non-duty days

Figure 3 illustrates work and sleep distributions for each location. For the EMS and SAR operations, work was spread across the day and night, with an average duty period length of 7.0 h (SD = 2.7 h). However, even in these operations, most work occurred during the daytime, and most sleep during night-time hours. The amount of sleep that occurred during duty time ranged from none in EMS in the UK, up to nearly 30% in Spain SAR. Sleep period length on duty (mean = 3.4 h, SD = 1.8 h) was shorter compared to off duty (mean = 6.5 h, SD = 0.3 h). However, there was also evidence of napping to complement shorter sleep periods taken during work, particularly in the Spain, France, and Italy operations. For the firefighting and transit operations, all duty periods occurred during the day, with an average duty period length of 8.5 h (SD = 1.8 h). Only 2–3% of sleep periods were associated with duty periods, and these had an average period length, 1.7 h (SD = 1.2 h). Average off-duty sleep period length was 7.0 h (SD = 0.4 h).

Total 24 h sleep time across all participants was significantly ($F_{1,3794.1}=138.6$, *p* < 0.001) shorter on duty days (EMM = 5.9 h, SE = 0.1 h) compared to non-duty days (EMM = 6.9 h, SE = 0.1 h).

For France EMS, subjective sleep quality ratings were significantly better ($F_{1,324.6}=5.3$, *p* = 0.022) for home sleep (EMM=2.6, SE=0.3) compared to sleep at the base (EMM = 2.8, SE = 0.3). However, ratings were average-good for both environments. For Spain SAR, subjective ratings indicated average-good sleep quality, and were not significantly different ($F_{1,154.1}=2.5$, *p* = 0.118) for home sleep (EMM = 2.9, SE = 0.2) compared to sleep at the base (EMM = 3.1, SE = 0.2). For UK Transfers, subjective ratings indicated average-good sleep quality, and were not significantly different ($F_{1,165.4}=3.1$, *p* = 0.085) when comparing home (EMM = 2.5, SE = 0.1) and hotel ratings (EMM = 2.8, SE = 0.2).

### Differences in work and sleep during peak and off-peak seasons

**Figure 4:**
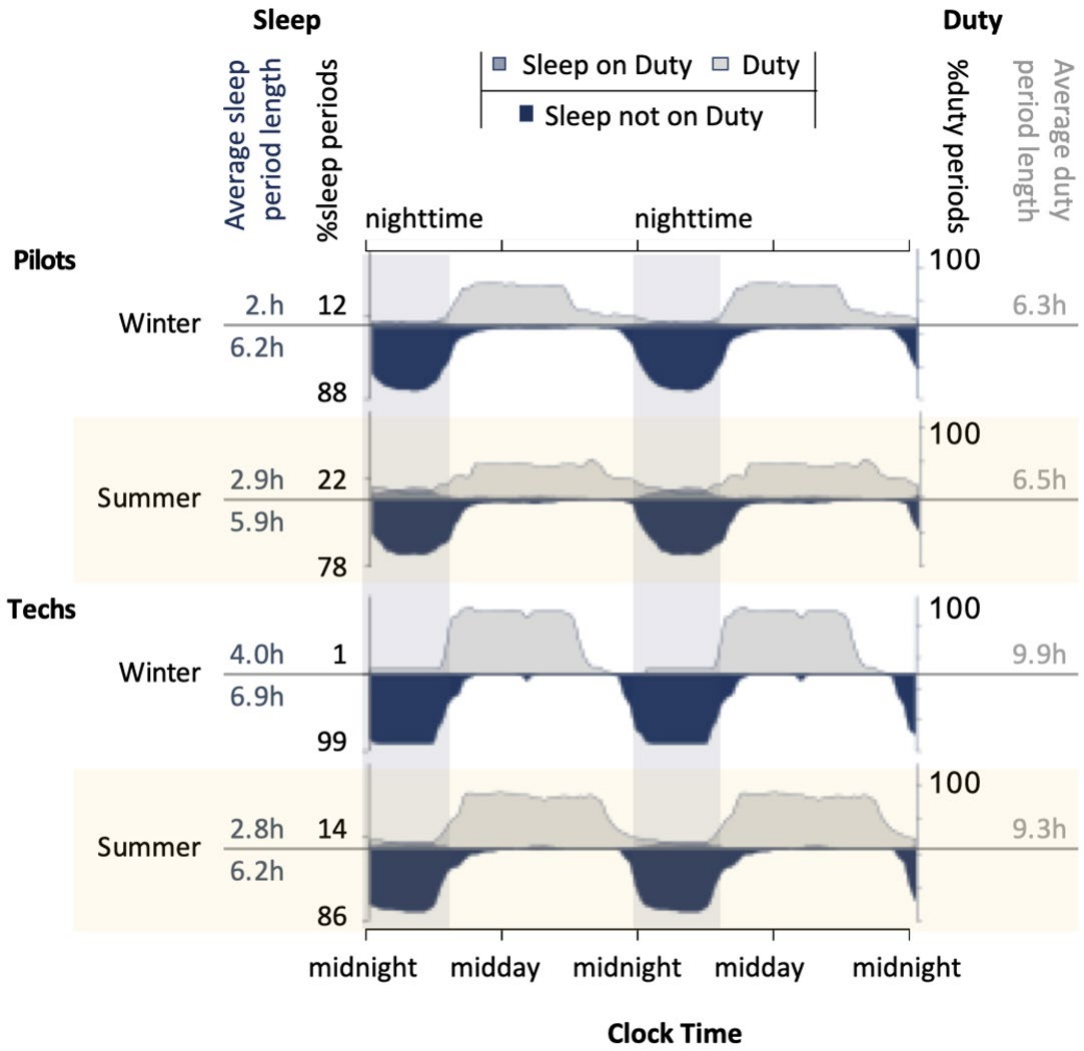
Work and sleep distributions vary as a function of season and job role for pilots and technicians (techs) in Italy. To characterise patterns of work and sleep, the 24 h distributions were double plotted. duty and sleep periods were expressed as a percentage of the total number of periods at that time of day (in 15-min bins). Sleep periods that were associated with duty were plotted, superimposed over duty, with sleep periods outside of duty also represented.

Figure 4 illustrates work and sleep distributions for Italy EMS pilots and technicians during winter and summer. During summer, duty periods are spread more over the day and night for both roles. In summer, for pilots, the proportion of sleep associated with duty increased from 12 to 22%, and for technicians the increase was from 1 to 14%. Technicians also supplemented sleep using afternoon naps in the winter, with a reduction in afternoon napping during the summer. For the Technicians, total sleep time in the 24 h prior to duty was significantly shorter ($F_{1,228.2}=26.7$, *p* < 0.001) in summer (EMM = 5.5 h, SE = 0.4 h) compared to winter (EMM = 6.8 h, SE = 0.4 h). For the pilots, there was no significant difference ($F_{1,206.9}=0.96$, *p* = 0.328) in summer (EMM = 5.8 h, SE = 0.3 h) compared to winter (EMM = 6.0, SE = 0.3 h).

The number of daylight hours in each location at each time of year was moderately correlated with the total sleep area under the curve ($r=-\,0.53$), such that as hours of sunlight per day increased, sleep decreased.

### Differences in sleep and wake as a function of work factors

**Figure 5:**
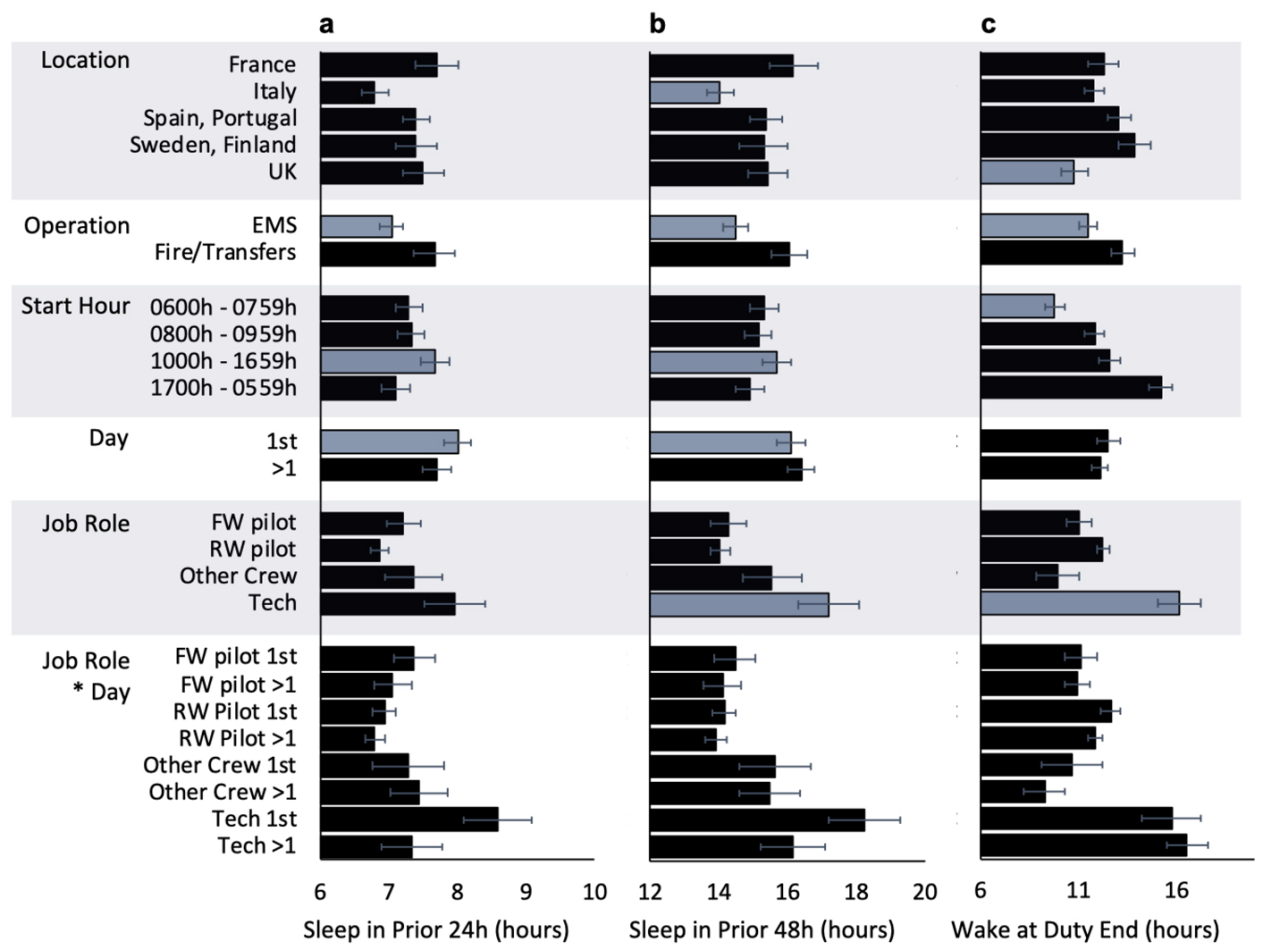
Sleep prior to duty and wake at duty end varies as a function of location, type of operation, shift start hour, consecutive days into duty, and job role. Figure displays estimated marginal means (bars) and their standard errors (whiskers) from the models conducted to examine differences in sleep in the 24 h (panel **a**) and sleep in the 48 h (panel **b**) prior to starting a duty period, and wake time at the end of duty (panel **c**) by work-related factors. Blue bars indicate significant differences from other levels of that variable, *p* < 0.05. *Note:* EMS = Emergency Medical Service, Fire = Firefighting, Transfers = Air Taxi, SAR = Search and Rescue, FW = Fixed Wing, RW = Rotary Wing, Tech = Technicians.

On average, total sleep time in the 24 h and 48 h prior to duty was highest in France and lowest in Italy (Fig. 5). The effect of location was not significant for sleep in the prior 24 h ($F_{4,109.7}=2.1$, *p* = 0.077). However, there was a significant effect of location for sleep in prior the 48 h ($F_{4,97.9}=2.7$, *p* = 0.035), such that sleep time was significantly shorter in Italy compared to the other locations (*p* < 0.05). There was also a significant effect of location for wake at duty end ($F_{4,93.7}=3.7$, *p* = 0.008), such that wake times at the end of duty were longer in Sweden and Finland than in the other locations (*p* < 0.05). There were significant differences between EMS and other operations for sleep in the prior 24 h ($F_{1,106.0}=6.6$, *p* = 0.012), sleep in the prior 48 h ($F_{1,93.7}=8.8$, *p* = 0.004), and wake at duty end ($F_{1,95.4}=8.5$, *p* = 0.004). Compared to Fire and Transfer operations, in Emergency Medical Services, on average, sleep in the 24 h, and 48 h prior to duty was significantly lower, and prior wake at the end of duty was significantly higher. There were significant differences by shift start hour for sleep in the prior 24 h ($F_{3,1097.6}=4.7$, *p* = 0.003), sleep in the prior 48 h ($F_{3,10891.0}=3.2$, *p* = 0.023), and wake at duty end ($F_{3,1014.9}=46.8$, *p* < 0.001). On average, duties starting during the day between 1000 and 1659 h were associated with higher amounts of prior sleep. Average wake times at the end of duty were significantly longer for duties starting later in the day. Duties starting between 0600 and 0759 h were associated with significantly shorter wake times than for any other start time. Average 24 h ($F_{1,1050.3}=5.7$, *p* = 0.018) and 48 h ($F_{1,1019.9}=7.1$, *p* = 0.008) sleep times were significantly longer prior to the first, compared to subsequent shifts in a sequence. There were no significant differences for prior wake at duty end ($F_{1,1073.4}=0.5$, *p* = 0.487). There was a significant effect of job role for sleep in the prior 48 h ($F_{3,102.1}=4.8$, *p* = 0.003) and wake at the end of duty ($F_{3,117.9}=7.5$, *p* < 0.001), such that compared to other job roles, technicians had the lowest sleep and the longest wake times. The effect of job role for sleep in the prior 24 h was not significant ($F_{3,120.6}=2.6$, *p* = 0.054). For technicians, sleep in the 24 h and 48 h prior to duty was longer before the first duty in a sequence, compared to subsequent duties. However, the day*job role interaction effect was not significant for sleep in the prior 24 h ($F_{3,1050.8}=2.6$, *p* = 0.053), sleep in the prior 48 h ($F_{3,1021.5}=2.4$, *p* = 0.066), or wake at duty end ($F_{3,1071.8}=0.6$, *p* = 0.647).

### Differences in PVT as a function of work factors

PVT Mean response times were, on average, 377 ms (SE = 1 ms). Overall, PVT mean response times were significantly longer on duty days compared to non-duty days ($F_{1,10853.1}=4.5$, *p* = 0.035), with a mean difference of 2 ms.

**Figure 6:**
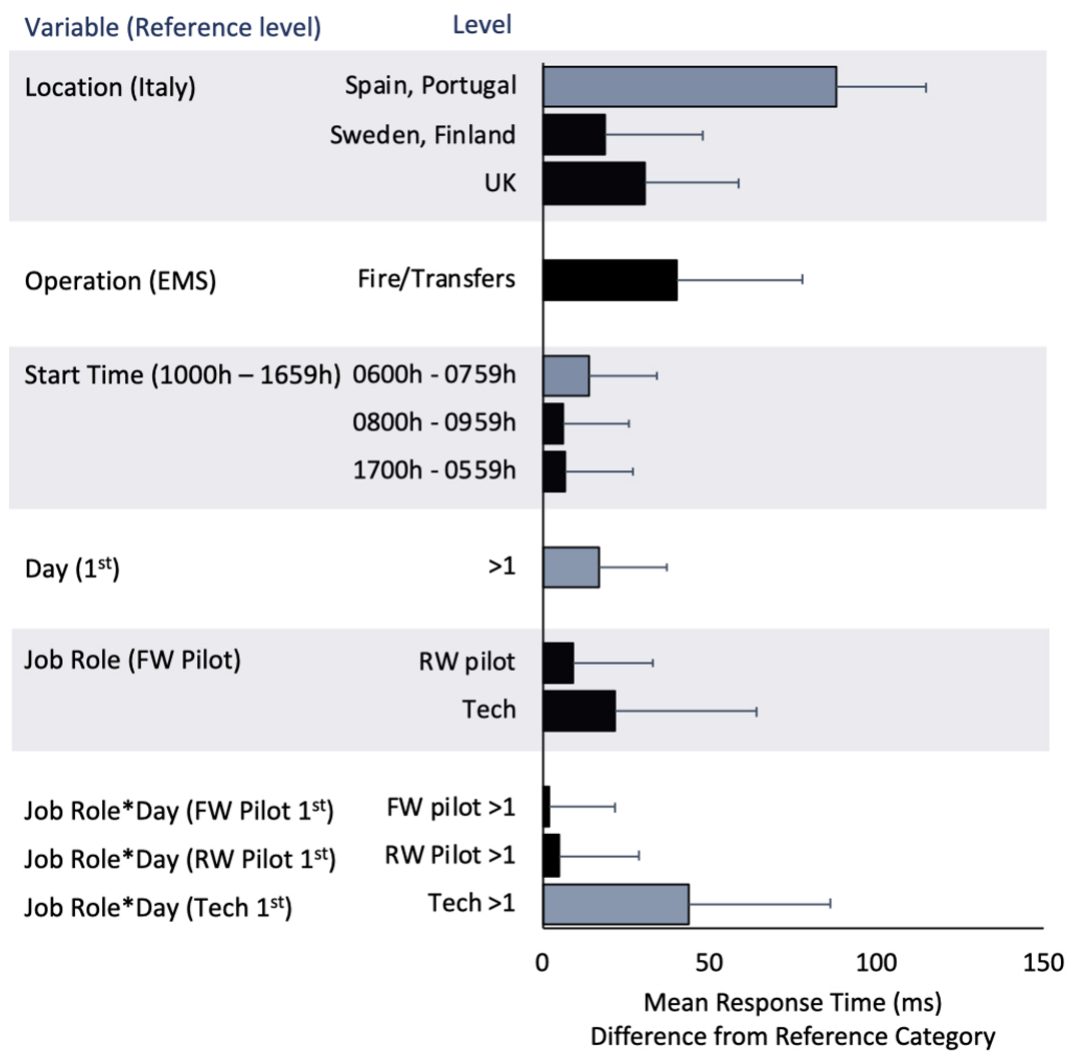
Psychomotor Vigilance Task (PVT) response times vary as a function of by location, shift start hour, consecutive days into duty, and job role. Differences in PVT mean response times (bars) and their standard errors (whiskers) area displayed relative to a reference level (e.g. for Location, differences in response times are shown relative to Italy). Blue bars indicate significant differences from other levels of that variable, *p* < 0.05.*Note:* EMS = Emergency Medical Service, Fire = Firefighting, Transfers = Air Taxi, SAR = Search and Rescue, FW = Fixed Wing, RW = Rotary Wing, Tech = Technicians.

There was a significant effect of location ($F_{4,70.2}=4.7$, *p* = 0.005), such that response times were significantly longer in Spain and Portugal, compared to Italy, Sweden and Finland, and the UK (*p* < 0.05), with differences between 69 and 88 ms (Fig. 6). There was a significant effect of shift start time on response times ($F_{3,2468.9}=6.9$, *p* < 0.001), such that shifts starting between 0600 and 0759 h were associated with significantly longer response times that those start at other times of day ($p<0.05$), with differences of 6–14 ms. The main effects of EMS/Other ($F_{1,67.0}=0.9$, *p* = 0.349) and job role ($F_{43,67.2}=0.1$, *p*=0.902) were not significant. There was a significant day*job role interaction effect ($F_{3,2442.5}=8.6$, *p* < 0.001), such that for technicians, response times on the first duty in a sequence were significantly shorter than those on subsequent duty days (*p* < 0.05), by an average of 44 ms.

### Examining lowest sleep and highest wake quintiles

**Figure 7:**
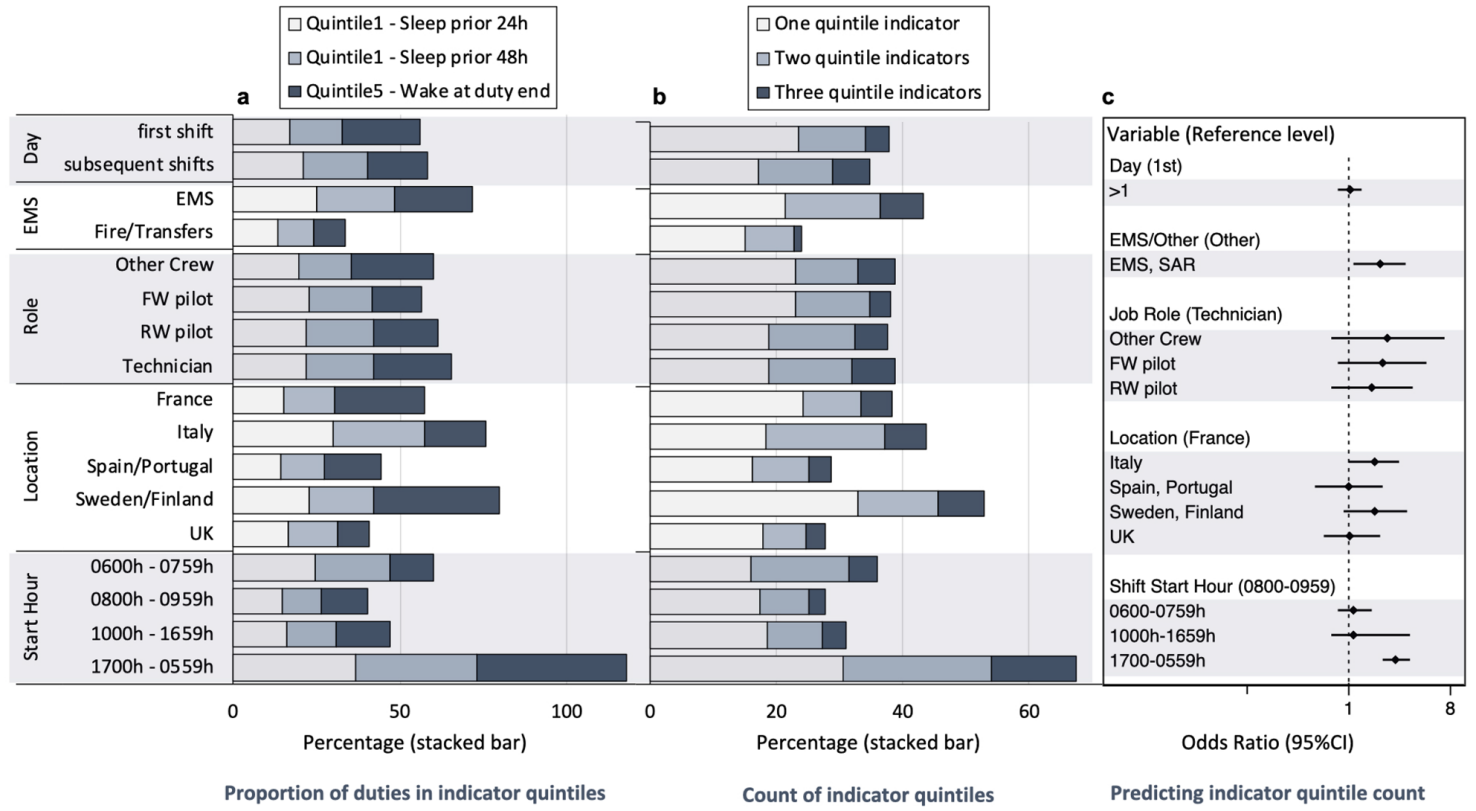
The odds of membership of multiple sleep and wake fatigue indicator quintiles (lowest quintiles for sleep in the prior 24 h and 48 h, and highest quintile for wake at duty end) are higher for EMS and SAR operations, and for duties starting in the evening and overnight. Stacked bar chart showing percentage of duties with prior sleep values in the lowest quintile and wake at the end of duty values in the highest quintile (panel **a**). Stacked bar showing percentage of duties that fall into one, two, or three indicator quintiles (panel **b**). Forest plot showing predictors of membership of multiple indicator quintiles for sleep and wake (0–3) by Location, Operation (EMS/SAR, Fire/Transfers), Shift Start Hour, Day, and Role (panel **c**). *Note:* EMS = Emergency Medical Service, Fire = Firefighting, Transfers = Air Taxi, SAR = Search and Rescue, FW = Fixed Wing, RW = Rotary Wing, Tech = Technicians.

The threshold value for the lowest quintile of sleep in the 24 h prior to duty was 6.0 h, and for sleep in the 48 h prior to duty it was 12.5 h. The threshold value for the highest quintile of wake at the end of duty was 14.2 h. The proportion of duties in each of the indicator quintiles is illustrated in Fig. 7 (panel a). Overall, 19.6% of duties were associated with one, 13.0% with two, and 5.2% with three thresholds, with the proportion of duties with one, two, or three indicators illustrated in Fig. 7 (panel b). The odds of multiple quintile indicators (7c) were significantly higher for those in EMS and SAR compared to firefighting and transfers (OR = 1.9; 95%CI = 1.1–3.2), and for duties starting across the evening and night (1700–0559 h) compared to daytime starts (0800–0959 h) (OR = 2.6, 95%CI = 2.0–3.5).

## Discussion

Across the diversity of aviation operations, locations, and roles in this study, most work occurred during daytime hours, and most sleep occurred at night. There was evidence of use of extended sleep and naps, particularly in the afternoons and on days off, to augment total sleep time. The proportion of sleep occurring during duty varied from zero to 30%. Sleep length and quality varied as a function of duty day (versus non-duty day), and location (home versus base). Sleep and wake also varied by operation, location, and role. Summer (peak season) was associated with work hours that were spread across the day, less sleep overall, and more sleep occurring during duty time. The odds of duties associated with the more than one sleep/wake fatigue indicator (as operationalised using the lowest quintiles of sleep in the 24 h and 48 h prior to starting a duty, and the highest quintile of wake at duty end) were significantly higher for those in emergency medical and search and rescue (24 h operations) relative to fire and transfers (daytime operations), and for duties that started in the evening or overnight compared to daytime starts. Technicians in Italy, who had the lowest recorded prior sleep and highest prior wake, showed evidence of prophylactic sleep to prepare for their long numbers of consecutive duty days. Overall, findings suggest that sleep at home and on base, in duty and non-duty time, may be used to compensate for aspects of duty schedules, including changes across seasons.

While the majority of sleep was observed during nighttime hours for the daytime operations (e.g. firefighting), perhaps surprisingly, the same was true for the 24 h operations. This may reflect that the nature of the 24-h services such as search and rescue and emergency medical response is to be available as required rather than working more continuously (not including breaks) such as with daytime firefighting. This kind of “on-call” space presents challenges for fatigue management, through the unpredictable nature of the work, and a lack of clarity as to how much sleep can be reasonably expected to occur in any period of opportunity and the quality of that sleep^43–45^. Sleep during duty periods has been documented in previous studies of rotary wing medical operations (e.g.^46^). As in the current study, increased sleep on off duty days relative to duty days has also been observed^24,27^. Work in long-haul airline pilots links increased sleep durations with lower recurrent on-duty sleepiness^20^.

Indeed, the finding that aviation personnel in such 24-h operations can get sleep, in particular in the on-call search and rescue and emergency medicine environments in this study, means that the opportunity exists for crew to maintain readiness for future tasks during work hours, as well as recover and prepare between duty periods. The importance of quality rest facilities to support sleep (the ability to lie down and limit noise and light) has been highlighted for emergency medical services^47^, as well as in in-flight rest-facility design for pilot sleep in commercial aviation^48,49^. In the current study, in 24-h operation locations there were generally facilities at the base that allowed sleep in private single bedrooms, often with private individual bathrooms, and with the ability to control temperature and lighting in the room. Such facilities were most frequently provided for pilots but were sometimes also accessible by people in other roles. Sleep otherwise could occur in base facilities including on reclining or lay-flat lounge/sofa chairs, on mattresses or on fold-out beds. Overall, sleep quality ratings were average to good across sleep environments, supporting the view that resourcing levels, rostering, workloads, and personal priorities related to sleep and fatigue management appear sustainable. For emergency medical service workers in France, quality ratings were lower for sleep on base compared to home sleep. Access to sleep during duty periods and access to facilities to support sleep at work is generally not assumed in legislation that regulates flight, duty, and minimum rest rules for aviation personnel. The use of on-duty sleep in these 24-h operations, and the variety of in sleeping environments, suggests potential benefits of methodical examination and customisation of on-base sleeping facilities.This argument can be extended to other “on-call” work environments, such as residential support^45^, or marine pilotage^50^, with such findings and regulatory innovations of potential interest beyond aviation operations.

Pilot on- and off-duty sleep strategies to compensate for work-related fatigue have not been systematically documented. A recent paper by Zaslona and colleagues analysed and described behavioural strategies that included use of in-flight rest and getting “lots of sleep” in order to reduce sleepiness and time awake when starting work^49^. In addition to the elevated sleep duration on non-duty days in the current study, there was evidence from sleep data, corroborated anecdotally by the Technicians in Italy, that due to their longer blocks of work they felt it was valuable to proactively prepare in advance of the first shift in a block. This is consistent with the idea of “banking sleep,”^51^ whereby extra sleep taken to prepare for upcoming sleep curtailment may be beneficial by virtue of the extinction of any existing sleep deficits^52,53^ There was also evidence from pilots and technicians in the current study of changes in sleep strategies as a function of season, in response to changes in duty patterns. Specifically, with longer daylight and work hours more spread across 24 h during peak season, pilots nearly doubled the proportion of sleep periods taken on duty (12–22%). Technicians did not sleep much at work in winter (1%), also increasing sleep during duty in summer (14%). Seasonal changes in work and sleep hours in rotary wing medical operations have been previously noted (e.g.^28^). The current study provides extra information about how sleep duration may change as a function of both seasonal duty patterns and compensatory behavioural response to these patterns. Taken together, these studies suggest that further research and documentation of compensatory behaviours for work-related fatigue, such as sleep “banking” and changing patterns in on-base, on-duty sleep, would be of benefit to further inform evidence-based FRMS guidelines and training to support the employee duty of care to be fit for work. Importantly, with the variation in sleep obtained during duty periods by operation and by season, changes to FRMS that currently assume sleep does not take place would not be altered to assume that it does. Rather, as in other environments where sleep can (but may not) occur at work, risk-based flexibility in guidelines would need to consider potential sufficiency of sleep (duration and quality) relative to the risks associated with the work task.

The relationship between shift timing, sleep, and wake was consistent with previous literature. Duties that started during the day (1000–1659 h) were associated with more sleep than duties starting at other times^54^ Sleep duration was longer prior to the first duty in a sequence, reflecting preparatory “banking”^49,55^. While these patterns resulted in the presence of work-related fatigue indicators, the relative incidence of multiple overlapping indicators was low. Emergency medical service personnel had less sleep in prior 24 and 48 h before a duty period on average but a shorter wake time at the end of duty periods. This is reflective of the nature of the task, which stops and starts more than in other roles such as firefighting. The intermittent nature of the work, permitting sleep during duty, to some extent balances the requirements for 24 h services from a fatigue likelihood perspective. This balance is akin to observations from the watchstanding and split sleep literature, that suggests the potential impact of circadian patterns of impairment may be mitigated by reducing impairment from prior sleep, long periods of wake, or both^56–60^.

Previous studies using reaction time tasks in aviation have found minimal differences across duties and schedules^25^, with converging evidence from other sources leading to conclusions that participants were not excessively fatigued in those operations^61^. In the current study Psychomotor Vigilance Task (PVT), response times were an average of 2 ms longer on work days compared to days off. With over 11,000 PVT observations, this very small difference was statistically significant. Compared to Italy, Spain and Portugal had the longest response times and the mean difference was 69-88 milliseconds. Consistent with previous research^54^, early duties (0600–0759 h) were associated with longer mean reaction times than those starting later in the day, consistent with reduced prior sleep as a function of waking early for work without the ability to advance bedtime due to the evening ’forbidden zone’ for sleep^62^. The only other difference in PVT response times was found for technicians who had PVT response times that were on average 44 ms shorter on their first duty day in a sequence than on subsequent duty days. This could reflect the preparatory sleep prior to their first duty. Unfortunately, due to the study design, we were unable to investigate response time patterns across multiple duties in more detail than a single comparison of the first with subsequent duties. The 21-day data collection did not include retrospective work history, so we were only able to identify first duties in a sequence if they occurred following one or more days off in the 21-day collection period (i.e. we were unable to identify how many duty days in a row participants had been working when data collection commenced). Building in this capacity in future studies would limit data exclusion and allow closer examination of patterns across duty sequences.

When considering differences in sleep and wake by operation and schedule-related factors in this study, it is important to acknowledge the limitations of the observational study design. Our dataset allows us to describe patterns in sleep and duty, but we do not have an indicator of how much time within each duty period (or rest period) was available for sleep, nor do we have data on other factors that influence sleep patterns. Taken together, the observations suggest that there are differences in sleep patterns that may be related to work type and seasonal differences in workload (supported by the correlation between work hours and daylight length). However, there are other factors that are likely playing a role that would be very beneficial to examine in future studies, including workplace culture and attitudes to sleep, and details of the sleep spaces provided. Similarly, in describing the patterns in PVT data, we do not have data on other factors that influence response times such caffeine consumption^63^. Like other candidates for fitness-for-work tests, PVT has been used in workplaces where caffeine consumption is not controlled^64,65^. In such circumstances, results may be conceptualised as an indicator of performance capacity at that point in time (which may be influenced by many factors). Nevertheless, it would have been beneficial for this study, if we had access to caffeine consumption data to help to put the PVT results in greater context, as has been done in other workplace studies^64^. Other limitations of the study include the lack of sufficient nighttime data, and the technical difficulties with the actigraphs as outlined in the Methods section.

Other areas of interest for further study that were beyond the scope of this work include the impact of operations without on-site sleeping accommodation, necessitating travel prior to duty^25,26^ and how sleep and napping are negotiated in single versus dual pilot operations, since research shows differential methods of organisation of on-duty sleep in multi-crew systems^49^. Indeed, systematic qualitative exploration and documentation of sleep strategies and how they differ as a function of location, day length, season, role, and shift design (e.g. number of consecutive duties, duty length, start times) would provide a highly beneficial depth and context to the patterns suggested by the large, quantitative snapshot taken in the current study.

Taken together, findings of this study suggest that rotary and fixed-wing aircraft operations in extreme environments, such as emergency medical and search and rescue may have critical similarities with other “on-call” environments that allow sleep at work and that differences in work design, and changes in environment and load (e.g. through seasonal rhythms) may trigger different compensatory patterns in sleep. These findings provide a useful focal point for fatigue risk management systems in aircraft operations in extreme environments, from consideration of individual behaviours through to legislation design.
